# Enhancing Transplant Awareness and Acceptance Through Targeting Knowledge Gaps and Common Misconceptions

**DOI:** 10.7759/cureus.52303

**Published:** 2024-01-15

**Authors:** Mohammed F Shaheen, Rawan Bukhari, Taif M Almutairi, Abdulrahman Altheaby, Abdulrahman Altamimi, Khalid Bin Saad

**Affiliations:** 1 Organ Transplant Center and Hepatobiliary Sciences Department, King Saud bin Abdulaziz University for Health Sciences, Riyadh, SAU; 2 College of Nursing, King Saud bin Abdulaziz University for Health Sciences, Riyadh, SAU; 3 King Abdullah International Medical Research Center, King Saud bin Abdulaziz University for Health Sciences, Riyadh, SAU

**Keywords:** public awareness campaign, misconceptions, knowledge gap, organ donation, transplant

## Abstract

Introduction

Organ transplantation is a critical intervention for patients with end-stage organ failure, but misconceptions and knowledge gaps often hinder organ donation. This study evaluates the acceptability and effectiveness of an organ donation campaign focusing on addressing knowledge gaps and misconceptions in Riyadh, Saudi Arabia.

Methods

A two-day awareness campaign was conducted in a shopping mall, featuring four stations providing information on various aspects of organ donation. Participants completed a self-administered, researcher-developed, questionnaire before and after the tour.

Results

Of the 201 participants, 167 completed the questionnaire (83% response rate). The majority (92.9%) reported learning new information and indicated that the knowledge improved their perspective on organ donation. A high percentage (93.5%) felt the campaign answered their questions, with 90.9% deciding to register as organ donors.

Conclusion

A knowledge-enhancing campaign can effectively improve public perception and promote awareness of organ donation and transplantation. However, the study is limited by its short timeframe, location, and subjective data. Future research should explore the impact of such campaigns on donor registrations and evaluate their effectiveness in different cultural contexts.

## Introduction

Organ transplantation is the only curative intervention available for patients suffering from end-stage organ failure. Although it is of great benefit to the recipient, the donation act remains charitable and does not generally harbor direct benefits to the donor [1]. Hence, the regulation of organ donation and transplantation has been a hot topic from an ethical, medical, and legislative standpoint for the past few decades [2]. Organ trafficking, as well as the organ black market, has been criminalized and fought against [3]. Nowadays, each society is responsible for securing the optimal medical care for those in need of organs while ensuring the ethical conduct of organ donations. Hence, organ donation awareness is now of utmost importance, particularly in societies that suffer a large gap between supply and demand for transplantable organs [4,5]. Although Saudi Arabia ranks high amongst the adjacent nations in terms of organ donation and transplantation, there continue to be recognizable issues that are affecting the general organ donation perception, awareness, and acceptance, particularly when it comes to donation after death [6-9]. In a recent publication, misconceptions surrounding brain death were noted as a major barrier preventing consent in Arab and Saudi donors [10].

Researchers have tried several promotional strategies to improve the rates of organ donation acceptance among their communities. One common approach has been to appeal to the emotions of the viewer through stories of patients and donors [11]. On the other hand, enhancing knowledge about a specific topic is a cornerstone for many public health campaigns such as campaigns about diabetes, smoking cessation, and depression or suicide [12-14]. Targeting the knowledge gaps in organ donation and transplantation campaigns may be difficult due to the inherited complexity of its concepts. Hence, it is not commonly adopted. Several studies have looked at the association between knowledge about donation and the willingness to donate or register as an organ donor. Repeatedly, willingness and acceptance of the idea of organ donation have been observed to be associated with better knowledge about the topic [9,15-18]. This raises the question if adopting a health promotional strategy that focuses on enhancing the knowledge about organ donation and transplantation in educational campaigns is effective.

In this study, we aimed to look at the acceptability and utility of an organ donation campaign that focused on tackling the knowledge gaps and widespread misconceptions about the topic as its main cornerstone. These gaps and misconceptions were identified through a pre-campaign literature search by the authors. Subsequently, a public organ donation and transplantation awareness campaign took place in a public shopping mall in Riyadh, Saudi Arabia, over two days to achieve this goal.

## Materials and methods

In January 2020, a two-day organ donation and transplantation awareness campaign was conducted in a large shopping mall in Riyadh, Saudi Arabia. The Institutional Review Board of King Abdullah International Medical Research Center, located in Riyadh, Kingdom of Saudi Arabia, approved the study (approval number: RYD-20-419812-109731).

**Figure 1:**
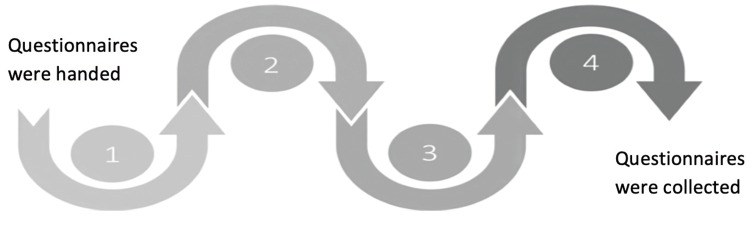
Campaign station arrangement: points of questionnaire distribution and collection

The campaign featured four sequential stations, each designed to provide information on a specific aspect of organ donation or address a common misconception. Public participation was voluntary, and data were collected through a self-administered paper questionnaire provided to participants at the beginning of their tour, which was collected upon completion of the activity (Figure 1).

At the first station, participants watched a three-minute video containing interviews with individuals who shared their experiences with living organ donation and transplantation (Figure 1A). This was followed by a factual presentation on the prevalence of organ failure in the community and the persistent gap between supply and demand. Next, participants moved to the second station, where a medical trainee explained the organ donation process for both deceased and living donors using low-cost models representing the body and various organs (Figure 1B). Here, participants learned about the liver's regenerative ability after donation and the sufficiency of a single kidney to meet the body's needs. In the third station, participants were presented with common misconceptions about organ donation and transplantation, which were then debunked and clarified (Figure 1C). These misconceptions included the reversibility of brain death, donor body mutilation, long-term harm to living donors, and Islamic religious views on organ donation and transplantation. Lastly, at the fourth station (Figure 1D), participants had the opportunity to ask a transplant physician and/or surgeon any further questions or concerns. The total time to complete all stations ranged from 10-15 minutes.

**Figure 2:**
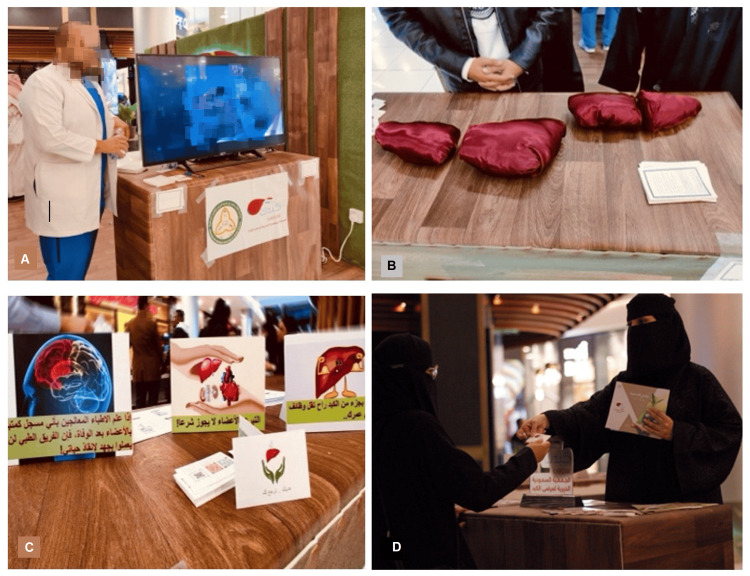
(A) Station 1, showing an illustrative three-minute video; (B) Station 2, explaining liver regeneration after donation; (C) Station 3, answering common misconceptions related to brain death, religious views, and the chance of organ failure after donation; (D) Station 4, answering participants' questions

The self-administered questionnaire had three parts. The first part, completed before participating in the stations, consisted of nine questions that aimed to gather basic demographic information and assess participants' knowledge and perception of organ donation. The second part, completed after the tour, contained eight questions designed to identify the most significant motivators for participants to accept organ donation based on their campaign experience and any barriers that might affect their willingness to donate. The third part of the questionnaire included six questions aiming to capture participants' opinions on the utility and acceptability of the campaign format and whether it provided them with new information about organ donation and transplantation. The last two parts of the questionnaire used a five-point Likert scale for scoring. Although administered in Arabic, a translated English version of the questionnaire is available in the supplementary materials (See Appendices).

Data were analyzed using IBM SPSS Statistics for Windows, Version 27.0 (Released 2020; IBM Corp., Armonk, New York, United States). After normality testing, continuous data were presented as means ± standard deviation, while categorical data were reported as absolute numbers and percentile proportions.

## Results

A total of 201 individuals aged 15 years and above participated in the campaign, with 167 of them completing all activities and submitting a filled questionnaire (83% response rate). The mean age of the participants was 27.2 ± 8.2 years, ranging from 15 to 63 years old. Males represented 61% of the participants. Additional sociodemographic variables are presented in Table 1. Notably, even though 35% of the participants reported knowing a friend or a family member who suffered end-stage organ failure requiring transplantation, 55% of participants reported having no or minimal understanding of organ donation and transplantation processes. While most participants (70%) initially held a positive perspective of organ donation, 5-8% of participants expressed a negative perspective towards organ donation (Table 1). The primary sources of information on the topic of organ donation and transplantation were reported to be the Internet (54%), healthcare personnel through personal interaction or campaigns (38%), and friends or family members (26%).

**Table 1:** Sociodemographic variables

Demographics	Values
Age, mean ± SD	27.2 ± 8.2 years
Male gender, n (%)	102 (61.1%)
Educational level	
Bachelor/Diploma, n (%)	81 (48.5%)
High school/Less, n (%)	48 (28.7%)
Master/PhD, n (%)	35 (21%)
Undeclared, n (%)	3 (1.8%)
Profession	
Studying, n (%)	55 (32.9%)
Working, non-healthcare related, n (%)	52 (31.1%)
Working, healthcare-related, n (%)	43 (25.8%)
Undeclared, n (%)	17 (10.2%)
Organ Donation Knowledge and Perception	
Self-Reported Understanding of Organ Donation and Transplantation Processes	
Good/excellent, n (%)	71 (42.5%)
Some/minimal, n (%)	56 (33.5%)
Almost none, n (%)	35 (21%)
Undeclared, n (%)	5 (3%)
Sources of Information Regarding Organ Donation and Transplantation	
Internet and social media, n (%)	90 (53.9%)
Doctors/awareness campaigns, n (%)	64 (38.3%)
Friends and relatives, n (%)	44 (26.3%)
Television programs, n (%)	34 (20.4%)
Newspapers, n (%)	18 (11.1%)
Formal education, n (%)	12 (7.2%)
Radio, n (%)	6 (3.6%)
No source, n (%)	24 (14.4%)
The Participant had Contemplated Registering as an Organ Donor	
No, n (%)	74 (44.3%)
Yes, but did not register, n (%)	58 (34.7%)
Yes, and registered, n (%)	30 (18%)
Undeclared, n (%)	5 (3%)
Initial Perspective Regarding Organ Donation	
Supportive, n (%)	117 (70.1%)
Neutral, n (%)	38 (22.8%)
Opposing, n (%)	8 (4.8%)
Undeclared, n (%)	4 (2.4%)

**Figure 3:**
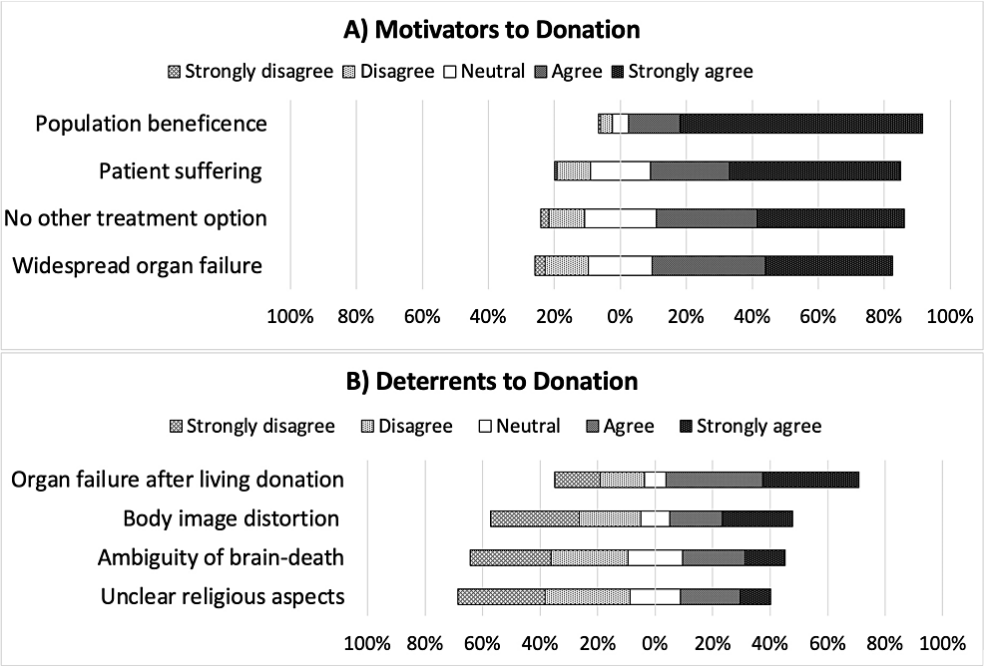
Motivators and deterrents to becoming an organ donor. The data has been represented as percentages (%).

After receiving information from the four stations, participants rated the most persuasive reasons for engaging in organ donation as well as reasons they perceived to constitute significant barriers. The most common reasons for willingness to donate were the sense of population beneficence (89.1%), witnessing patient suffering (75.9%), the lack of alternative treatment options for patients with end-stage organ failure (75.3%), and the widespread prevalence of organ failure (72.8%) (Figure 3A). Conversely, the most common barriers affecting donation willingness were the fear of organ failure after living donation (66.2%), concern about body image distortion after deceased donation (42.5%), the ambiguity surrounding the concept of brain death (35%), and conflicting religious opinions and perspectives (31.2%) (Figure 3B).

Upon completing the campaign, the vast majority of participants (92.9%) reported learning new information about organ donation. All of those reported that the newly acquired knowledge further improved their perspective toward organ donation. Furthermore, almost all participants (93.5%) felt that the campaign answered all their questions about organ donations, which encouraged 90.9% of them to support donation efforts by deciding to register as organ donors in the national registry. Interestingly, five of the eight participants who initially held a negative perception of organ donation reported considering registering as an organ donor by the end of the campaign.

## Discussion

The strategy of publicly addressing factual knowledge and correcting misconceptions to encourage organ donation is uncommon, particularly in the Middle East, where the complex interplay of cultural and religious factors surrounding the topic can potentially complicate the conversation. Although organ donation is endorsed as a religiously charitable act by the major religious bodies in the region; misconceptions regarding Islamic views continue to affect the public’s willingness to donate [19]. Several studies examining attitudes and perceptions towards organ donation have identified a lack of sufficient knowledge on the subject as a barrier as well as found a positive correlation between the level of knowledge about organ donation and willingness to donate [20-23]. Moreover, studies on related subjects indicate the potential advantages of integrating knowledge-enhancing strategies into health education campaigns focused on organ donation.

Few available studies implying public knowledge-promoting strategies in organ donation have demonstrated positive outcomes when implemented. For instance, a Greek study employed an interactive online questionnaire to assess perceptions and attitudes toward cornea donation while educating participants on the process and value of cornea donation. The study revealed that improved knowledge significantly influenced a favorable change in attitudes toward cornea donation and increased willingness to become a cornea donor among the Greek population [24]. Another study conducted in London, Ontario, Canada, involved a pilot educational campaign to inform attendees of junior hockey league games about deceased organ donation. During the campaign, a modest increase in donor registrations was observed [25]. To our knowledge, there is no published data examining the utility of similar educational campaigns in the field of organ transplantation in our region.

This study primarily highlights the acceptability of the approach among the population when executed appropriately. Despite the campaign's reliance on factual and scientific narratives accompanied by straightforward models for aiding explanation and visualization for non-medical individuals, participants' responses were predominantly positive. It is important to note that this outcome was achieved within a culture that tends to be reserved and unaccustomed to such an approach, especially concerning a sensitive topic like organ donation.

The questionnaire asked the participants to declare their opinions before and then after participation in the campaign, revealing an improvement in perceptions. Eight participants engaged in the campaign with initial negative perceptions about organ transplantation. It was interesting to have five of them change their views by the end of the campaign to the extent that they reported their intention to register as organ donors. This, we believe, is reflective of the potential impact of the strategies followed in the educational campaigns.

Although the results are encouraging, our study remains limited by the short timeframe, the number of participants, and the fact that it takes place in one location in Riyadh. As this was a researcher-made poll, all the data provided are subjective. For example, there are no objective numbers about how many people who joined or registered to be organ donors. Additionally, the voluntary nature of participation may have selectively attracted those who had a positive attitude or knowledge about the topic.

## Conclusions

Enhancing the public’s knowledge about organ donation and transplantation through focused education incorporated with clear messages improves their perception and represents a successful strategy to promote awareness of organ donation and transplant. However, the study is limited by its short timeframe, location, and subjective data. Future research should explore the impact of such campaigns on donor registrations and evaluate their effectiveness in different cultural contexts.

## Figures and Tables

**Figure 4:**
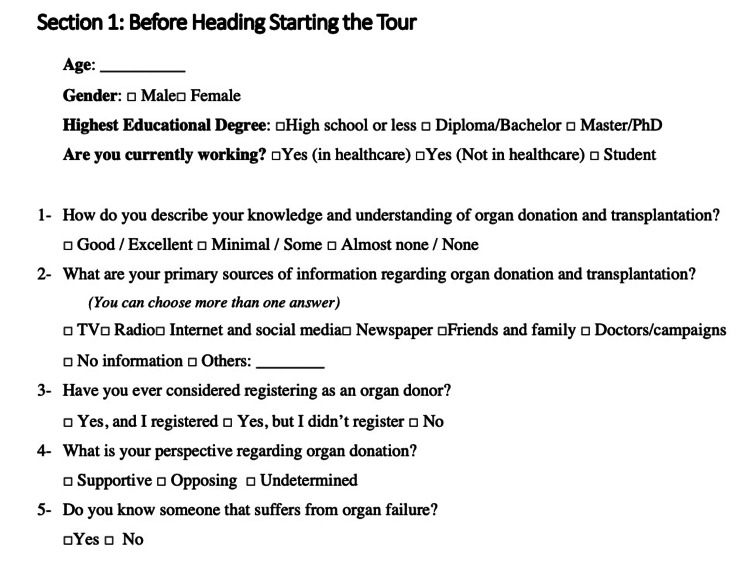
Questionnaire Page 1

**Figure 5:**
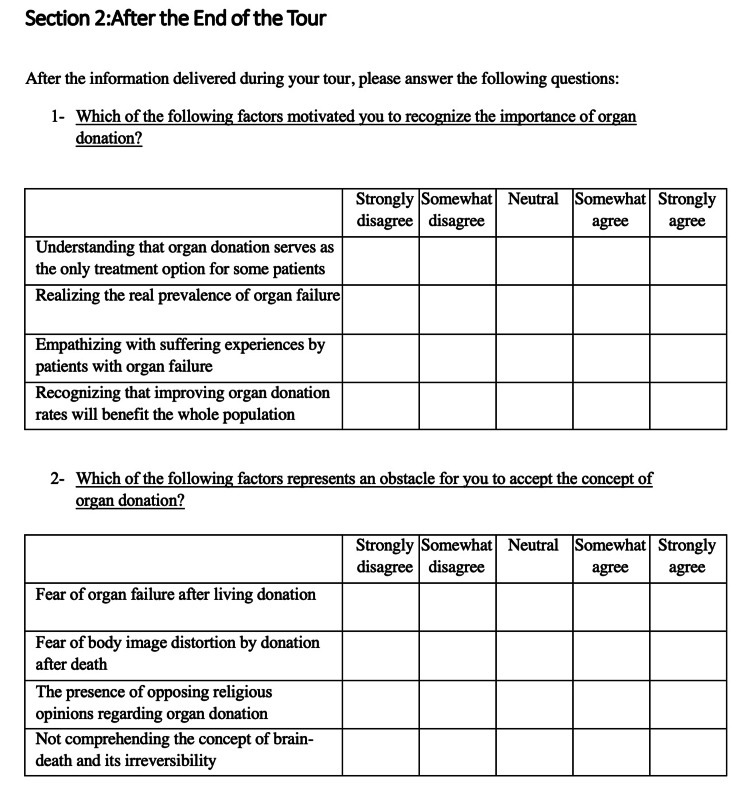
Questionnaire Page 2

**Figure 6:**
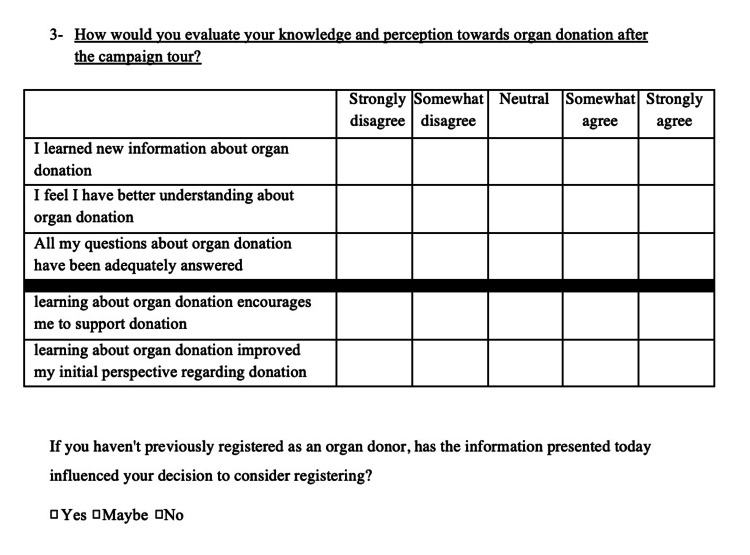
Questionnaire Page 3

## References

[1] Glazier AK (2011). The principles of gift law and the regulation of organ donation. Transpl Int.

[2] Iltis AS, Rie MA, Wall A (2009). Organ donation, patients' rights, and medical responsibilities at the end of life. Crit Care Med.

[3] Danovitch GM, Chapman J, Capron AM (2013). Organ trafficking and transplant tourism: the role of global professional ethical standards-the 2008 Declaration of Istanbul. Transplantation.

[4] Simillis C (2010). Do we need to change the legislation to a system of presumed consent to address organ shortage?. Med Sci Law.

[5] Levitt M (2015). Could the organ shortage ever be met?. Life Sci Soc Policy.

[6] Al-Sebayel MI, Al-Enazi AM, Al-Sofayan MS (2004). Improving organ donation in Central Saudi Arabia. Saudi Med J.

[7] Aldawood A, Al Qahtani S, Dabbagh O, Al-Sayyari AA (2007). Organ donation after brain-death: experience over five-years in a tertiary hospital. Saudi J Kidney Dis Transpl.

[8] Shaheen FA, Souqiyyeh MZ (2004). Increasing organ donation rates from Muslim donors: lessons from a successful model. Transplant Proc.

[9] Alsharidah DS, Al-Dossari FS, AlMahmoud N (2018). Assessment of knowledge and attitude toward organ donation among the Saudi population in Riyadh City. Saudi J Kidney Dis Transpl.

[10] Shaheen MF, Alghafees MA, Alzeer ZA, Altheaby A, Shaheen FA (2023). Barriers to deceased donor procurements: the Saudi experience. Transplantation.

[11] Thornton JD, Patrick B, Sullivan C (2019). Comparing web-based video interventions to enhance university student willingness to donate organs: a randomized controlled trial. Clin Transplant.

[12] Holder M, Ehehalt S (2020). Significant reduction of ketoacidosis at diabetes onset in children and adolescents with type 1 diabetes-the Stuttgart Diabetes Awareness Campaign, Germany. Pediatr Diabetes.

[13] Duke JC, Woodlea R, Arnold KY, MacMonegle AJ, Nonnemaker JM, Porter L (2020). Effect of a statewide media campaign on smoking cessation among Florida adults. Prev Chronic Dis.

[14] Dumesnil H, Verger P (2009). Public awareness campaigns about depression and suicide: a review. Psychiatr Serv.

[15] Agrawal S, Binsaleem S, Al-Homrani M, Al-Juhayim A, Al-Harbi A (2017). Knowledge and attitude towards organ donation among adult population in Al-Kharj, Saudi Arabia. Saudi J Kidney Dis Transpl.

[16] Al Bshabshe AA, Al-Ghamdi BA, Habash S, Al-Harthi M, Dwaima M, Wani JI (2018). secondary school students' orientation toward brain death and organ donation in southern region of Saudi Arabia. Transplant Proc.

[17] Sayedalamin Z, Imran M, Almutairi O, Lamfon M, Alnawwar M, Baig M (2017). Awareness and attitudes towards organ donation among medical students at King Abdulaziz University, Jeddah, Saudi Arabia. J Pak Med Assoc.

[18] AlShareef SM, Smith RM (2018). Saudi medical students knowledge, attitudes, and beliefs with regard to organ donation and transplantation. Saudi J Kidney Dis Transpl.

[19] Krupic F (2020). The impact of religion and provision of information on increasing knowledge and changing attitudes to organ donation: an intervention study. J Relig Health.

[20] Abdulrazeq F, Matsumoto MM, Zourob M (2020). Barriers in knowledge and attitudes regarding organ donation among Urban Jordanian population. Saudi J Kidney Dis Transpl.

[21] Hajjar WM, Bin Abdulqader SA, Aldayel SS, Alfardan AW, Alzaidy NI (2016). Knowledge, attitudes, and beliefs toward organ donation among social media users. Transplant Proc.

[22] Khushaim L, Al Ghamdi R, Al-Husayni F, Al-Zahrani A, Khan A, Al Zahrani A (2018). The knowledge, perception and attitudes towards organ donation among general population - Jeddah, Saudi Arabia. Int J Adv Res.

[23] Iliyasu Z, Abubakar IS, Lawan UM, Abubakar M, Adamu B (2014). Predictors of public attitude toward living organ donation in Kano, northern Nigeria. Saudi J Kidney Dis Transpl.

[24] Tsigkos D, Tzelepi A, Kopsini D, Manolakou D, Konistis E, Palioura S (2020). Interactive online survey raises awareness about cornea donation. BMJ Open Ophthalmol.

[25] Naylor KL, McKenzie S, Cherry C (2017). Evaluation of a hockey deceased organ donation awareness campaign: a population-based cohort study. Can J Kidney Health Dis.

